# Autoantibodies Neutralizing GM-CSF in HIV-Negative Colombian Patients Infected with *Cryptococcus gattii* and *C. neoformans*

**DOI:** 10.1007/s10875-024-01757-y

**Published:** 2024-07-15

**Authors:** Carlos A. Arango-Franco, Julian Rojas, Carolina Firacative, Mélanie Migaud, Clara Inés Agudelo, José Luis Franco, Jean-Laurent Casanova, Anne Puel, Jairo Lizarazo, Elizabeth Castañeda, Andrés A. Arias

**Affiliations:** 1 Group of Inborn Errors of Immunity (Primary Immunodeficiencies), Department of Microbiology and Parasitology, School of Medicine, University of Antioquia (UdeA), Medellín, Colombia; 2 Laboratory of Human Genetics of Infectious Diseases. Necker Branch, INSERM U1163, Necker Hospital for Sick Children, Paris, France; 3 Studies in Translational Microbiology and Emerging Diseases (MICROS) Research Group, School of Medicine and Health Sciences, Universidad del Rosario, Bogotá, Colombia; 4 Microbiology Group, Instituto Nacional de Salud, Bogotá, Colombia; 5 University of Paris Cité, Imagine Institute, Paris, France; 6 St. Giles Laboratory of Human Genetics of Infectious Diseases, Rockefeller Branch, The Rockefeller University, New York, NY USA; 7 Howard Hughes Medical Institute, New York, NY USA; 8 Pediatric Hematology and Immunology Unit, Necker Hospital for Sick Children, Paris, France; 9 Internal Medicine Department, Hospital Universitario Erasmo Meoz, University of Pamplona, Cúcuta, Colombia; 10 School of Microbiology, University of Antioquia (UdeA), Medellin, Colombia

**Keywords:** Cryptococcosis, Granulocyte-macrophage colony-stimulating factor (GM-CSF), *Cryptococcus neoformans*, *Cryptococcus gattii*, Anti-cytokine autoantibodies, Human immunodeficiency virus (HIV), Neutralizing auto-abs against GM-CSF

## Abstract

**Background:**

Cryptococcosis is a life-threatening disease caused by *Cryptococcus neoformans* or *C. gattii*. Neutralizing autoantibodies (auto-Abs) against granulocyte-macrophage colony-stimulating factor (GM-CSF) in otherwise healthy adults with cryptococcal meningitis have been described since 2013. We searched for neutralizing auto-Abs in sera collected from Colombian patients with non-HIV-associated cryptococcosis in a retrospective national cohort from 1997 to 2016.

**Methods:**

We reviewed clinical and laboratory records and assessed the presence of neutralizing auto-Abs against GM-CSF in 30 HIV negative adults with cryptococcosis (13 caused by *C. gattii* and 17 caused by *C. neoformans*).

**Results:**

We detected neutralizing auto-Abs against GM-CSF in the sera of 10 out of 13 (77%) patients infected with *C. gattii* and one out of 17 (6%) patients infected with *C. neoformans*.

**Conclusions:**

We report eleven Colombian patients diagnosed with cryptococcosis who had auto-Abs that neutralize GM-CSF. Among these patients, ten were infected with *C. gattii* and only one with *C. neoformans.*

**Supplementary Information:**

The online version contains supplementary material available at 10.1007/s10875-024-01757-y.

## Key Points

• The importance of anticytokine autoantibodies in the pathogenesis of various infectious diseases, including opportunistic mycoses, is increasingly being reported.

• Neutralizing auto-Abs against GM-CSF are present in Colombian patients with cryptococcosis caused by *C. gattii* or *C. neoformans.*

**Supplementary Information:**

The online version contains supplementary material available at 10.1007/s10875-024-01757-y.

## Introduction

Cryptococcosis begins with the inhalation of dehydrated yeast cells or basidiospores of the two species complexes *Cryptococcus neoformans* or *C. gattii*, typically originating from soil, avian excreta, trees, or decaying wood [1]. Cryptococcal disease initially presents as pneumonia and later disseminates to the central nervous system (CNS), causing meningoencephalitis [1]. Despite common environmental exposure to cryptococcal species, cryptococcosis rarely occurs in the healthy population due to high natural resistance. Defects of T-cell-mediated immunity, specifically a decrease in the number and/or the function of CD4^+^ lymphocytes, as seen in human immunodeficiency virus (HIV)-infected individuals, remain the main risk factor for acquiring *C. neoformans*-induced cryptococcosis [1]. Cryptococcosis caused by *C. gattii*, which is much less common (~ 20%), has traditionally been considered to occur in otherwise healthy individuals, particularly HIV-seronegative, or those with unknown risk factors [2]. However, immunosuppression other than HIV or pulmonary diseases can be associated with greater risk for *C. gattii* infection [2]. Moreover, host-dependent risk factors have been detected in most patients with cryptococcosis caused by *C. gattii*, suggesting that this specie is an opportunistic pathogen [3, 4].

Neutralizing autoantibodies (auto-Abs) against specific cytokines are considered as autoimmune phenocopies of inborn errors of immunity (IEI) with a selective predisposition to infectious diseases [5–7]. Indeed, neutralizing auto-Abs against interleukin (IL)-17 A/F can cause chronic mucocutaneous candidiasis, those against IL-6 can lead to recurrent staphylococcal skin diseases, and those against type I IFNs can result in severe viral diseases such as critical COVID-19, influenza, Middle East Respiratory Syndrome (MERS) pneumonia, West Nile virus (WNV) encephalitis, or severe yellow fever virus (YFV) vaccine disease [5, 7, 8]. Lastly, neutralizing auto-Abs against interferon-gamma (IFN-γ) can lead to adult-onset susceptibility to diseases caused by intramacrophagic microbes, such as mycobacteria. They have also been reported in rare cases of HIV-negative patients with cryptococcosis [9, 10].

High titers of neutralizing auto-Abs against granulocyte-macrophage colony-stimulating factor (GM-CSF) were first described in adult patients with idiopathic pulmonary alveolar proteinosis (PAP), a severe lung disease characterized by the accumulation of surfactants in the alveoli, progressive respiratory failure, and an increased risk of secondary infections [11]. PAP in these patients can be isolated or associated with pulmonary or extrapulmonary infectious diseases, caused by various pathogens, including *Nocardia* spp., *Cryptococcus* spp., *Mycobacterium* spp., *Histoplasma* spp., or *Aspergillus* spp. [12]. High titers of neutralizing auto-Abs against GM-CSF have also been identified in patients with adult-onset isolated idiopathic disseminated diseases, mostly cryptococcosis, almost exclusively caused by *C. gattii* [12–15], nocardiosis, or, more rarely, aspergillosis [12, 14]. The causal relationships between the presence of neutralizing auto-Abs against GM-CSF and the two clinical phenotypes (PAP and cryptococcosis) are not fully understood. Nevertheless, patients with such auto-Abs first presenting with cryptococcosis have been reported with or without PAP manifestations, and patients first identified with PAP have been described with or without cryptococcosis [13]. Altogether, the presence of auto-Abs against GM-CSF in these pathologies, suggests an important role of GM-CSF in the correct maturation and function of alveolar macrophages, which represent the main cellular component of immunity against *Cryptococcus* [16, 17].

Given that approximately 13% of cryptococcosis cases in Colombia occur in HIV negative patients without apparent risk factors [18, 19], and considering the recent identification of neutralizing auto-Abs against GM-CSF in three Colombian patients with cryptococcal meningitis [20], we tested the hypothesis that neutralizing auto-Abs against GM-CSF may underlie cryptococcosis in other seemingly healthy Colombian individuals. Therefore, this study aimed to assess the presence of auto-Abs against GM-CSF in the serum of 30 HIV-negative Colombian patients who developed cryptococcosis caused by *C. gattii* or *C. neoformans* species complexes, and to correlate these findings with the patients’ clinical data.

## Materials and Methods

### Selection of Subjects and Sera

Between 1997 and 2016, as part of the National Surveillance Program for *Cryptococcus* and cryptococcosis in Colombia, led by the Instituto Nacional de Salud, in Bogotá, 1974 surveys of patients with cryptococcosis were completed. These surveys contain demographic data, risk factor information, clinical manifestations, diagnostic methods, and the patients’ initial treatment [18]. Considering that the survey does not include a follow-up of the patients, we focused exclusively on the clinical data that were collected at the moment of the survey. From these surveys, 392 (19.9%) patients were diagnosed as HIV negative; among these, the etiological agent of cryptococcosis was identified in 343 cases: 292 (85.1%) caused by *C. neoformans* and 51 caused by *C. gattii* (14.9%) [18] (Supplementary data, survey description). Serum samples were collected from 30 HIV negative patients with cryptococcosis over a 15-year period, from 1997 to 2011, and stored in the serum collection of the Microbiology Group at the Instituto Nacional de Salud. Among them, 13 were from patients infected with *C. gattii*, and 17 were from patients infected with *C. neoformans* [18] (Supplementary Tables 1, 2 and 3).

As part of the diagnosis of cryptococcosis, direct visualization of the encapsulated yeast cells in cerebrospinal fluid (CSF) using India ink was performed. For some of the sera, data on the titer of the cryptococcal antigen (CrAg) were available. CrAg titers were also measured in some patients’ CSF. All isolates of *C. gattii* or *C. neoformans* recovered from the studied patients were identified by routine phenotypic methods. Most *C. gattii* isolates had data on antifungal susceptibility, serotype, mating type, molecular type, and sequence type (ST), while most of *C. neoformans* isolates had data on serotype, mating type and molecular type [18, 19, 21] (Supplementary Tables 4 and 5). In addition, all studied sera had data on total levels of IgG, IgA and IgM, as well as cryptococcal-specific IgG, IgA and IgM as previously established [22]. For some analyses, data on specific levels of serum immunoglobulins against cryptococcal proteins were obtained from healthy adults without cryptococcosis or any other infectious disease (healthy controls) [22] (Supplementary Fig. 1).

### Detection of Anti-cytokine Auto-Abs by Multiplex particle-Based Assay

BD Cytometric Bead Array (BD CBA Flex system) were coated with 10 µg of recombinant human cytokine (IFN-α, IFN-β, IFN-ω, IFN-γ, IL-12p40, IL-17A, IL-23, IL-6 and GM-CSF - Biotechne) according to the manufacturer’s instructions (BD 558556). After validation of the coupling, the beads were incubated for 2 hours with serum samples from the 30 patients with cryptococcosis, positive controls, or healthy donors (1/10 000e dilution in PBS 1 × 2% BSA). After washing twice with PBS 1 × 0.005% Tween, the beads were incubated with a PE goat anti-human IgG antibody (SouthernBiotech C3923-S083E). Two washes in PBS 1 × 0.005% Tween were then performed. Finally, the beads were acquired on an Agilent Novocyte NovoSampler Pro, and data were analyzed using the FlowJo software v.10.6.2 (Becton Dickinson).

### Detection of Neutralizing Auto-Abs Against GM-CSF by Flow Cytometry

We tested whether the auto-Abs against GM-CSF could neutralize its activity and block STAT5 phosphorylation in human peripheral blood mononuclear cells (PBMCs). PBMCs were isolated from whole blood of a healthy donor by Ficoll-Hypaque density centrifugation (Amersham-Pharmacia- Biotech). The cells were counted and plated at 2 × 10^6^ cells/well in 96-well V-bottom plates (Thermo Fisher Scientific) in 100 µL of RPMI (Gibco BRL, Invitrogen) supplemented with 10% fetal bovine serum (Gibco BRL, Invitrogen), or 100 µL of RPMI supplemented with 1:10 serum from patients or controls. The PBMCs were either left unstimulated or stimulated with 10 ng/mL of rhIL-3 or 50 ng/mL of rhGM-CSF (Miltenyi-Biotec) for 15 min at 37 °C. Thereafter, the cells were fixed and permeabilized with a fixation/permeabilization kit (eBioscience). Extracellular labeling was performed with antibodies anti CD14-Pacific Blue and anti CD4-FITC (Sony-Biotechnology, clones M5E2 and RPA-T4, respectively). Cell viability was determined with the Aqua Dead Cell Stain Kit (Thermo-Fisher-Scientific). The level of phosphorylated STAT5 (p-STAT5) was assessed via intracellular staining with a Phospho-Flow PE Mouse Anti-p-STAT5 (pY694) antibody (BD Biosciences) in CD14^+^ cells. The data were collected with a Gallios flow cytometer (Beckman-Coulter) and analyzed with FlowJo software v.10.6.2 (Becton–Dickinson) (Supplementary Fig. 2).

### Statistical Analysis and Data Availability

The p-value was calculated among groups with a chi-square test with Fisher’s correction (given the low n in some of the cells in contingency tables). A p-value < 0.05 was considered to indicate statistical significance. All the raw and processed data will be made available by the corresponding authors upon request.

## Results

### Cryptococcosis in Colombian HIV Negative Patients

We studied 30 HIV negative unrelated Colombian patients with cryptococcosis caused by *C. gattii* (P1 to P13) (*n* = 13) or *C. neoformans* (P14 to P30) (*n* = 17). The demographic and clinical characteristics of the 30 studied patients are summarized in the supplementary material (Supplementary tables 1, 2 and 3). One patient (P28) also developed pulmonary tuberculosis (Tb) caused by *Mycobacterium tuberculosis* (*Mtb*) one year after being diagnosed with disseminated cryptococcosis due to *C. neoformans*. Among the patients, twenty-one (70%) were men, and nine (30%) were women, ranging from 1 to 71 years old, with an average age of 40.8 years. While most patients (86.7%) did not have any recognizable predisposing factors, hematological malignancies were detected in two patients (6.7%), and systemic lupus erythematosus (SLE) and rheumatoid arthritis in one patient each (3.3%). Although signs and symptoms of cryptococcosis varied among the studied patients, headache (70%), mental confusion (46.7%), and nausea (40%) were the most frequent clinical manifestations, which were expected considering that most patients (96.7%) were diagnosed with cryptococcal meningitis. None of the patients studied had a history of PAP at the time of inclusion. Treatment was mainly with amphotericin B deoxycholate alone (46.7%), or in combination with fluconazole (30%). Outcomes were recorded for 11 patients, eight of whom recovered after treatment, while three died of cryptococcosis [18].

### Auto‑Abs Against GM-CSF in Patients with Cryptococcosis

**Fig. 1:**
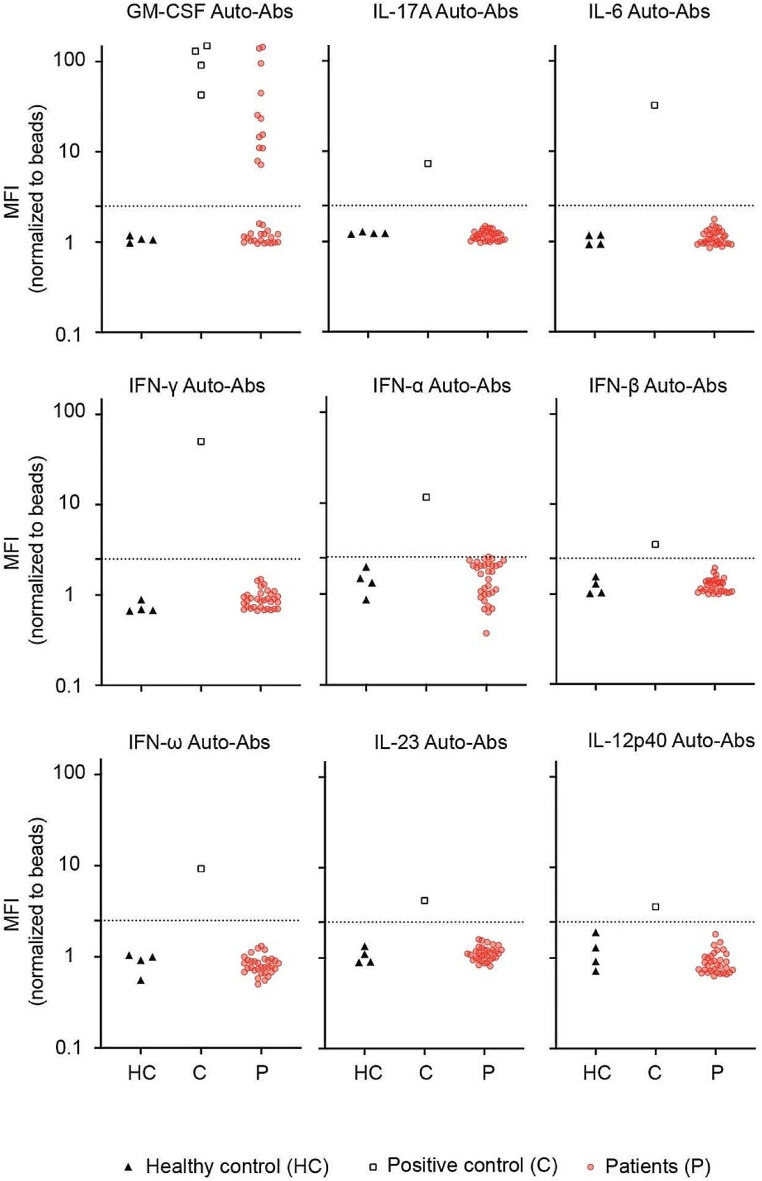
Detection of IgG auto-Abs in patients with cryptococcosis. Multiplex particle-based assay for auto-Abs against GM-CSF, IL-17 A, IL-6, IFN-α, IFN-β, IFN-γ, IFN-ω, IL-23, and IL-12p40 in serum from patients with cryptococcosis (*n* = 30) (P1 to P30), healthy controls (*n* = 4), and a patient positive for auto-Ab against each cytokine. HC, healthy controls; C, positive controls for each cytokine; P, patients with cryptococcosis

We searched for the presence of auto-Abs against nine cytokines (IFN-α, IFN-β, IFN-ω, IFN-γ, IL-12p40, IL-17 A, IL-23, IL-6 and GM-CSF) in the serum samples of the 30 patients with cryptococcosis. Multiplex particle-based flow cytometry revealed a high fluorescence intensity (MFI) for IgG auto-Abs against GM-CSF in the serum of 12 patients (Fig. 1). Ten of these 12 patients were infected by *C. gattii*, whereas 2 were infected by *C. neoformans*. In contrast, no auto-Abs directed against type I (IFN-α, IFN-ω and IFN-β) or type II (IFN-γ) interferons, IL-12p40, IL-17 A, IL-23 and IL-6 were detected in patients’ serum samples (Fig. 1). Taken together, these findings suggest that the presence of auto-Abs against GM-CSF predisposed these 12 otherwise healthy individuals to develop cryptococcosis.

### The Auto-Abs Neutralize GM-CSF in Vitro

**Fig. 2:**
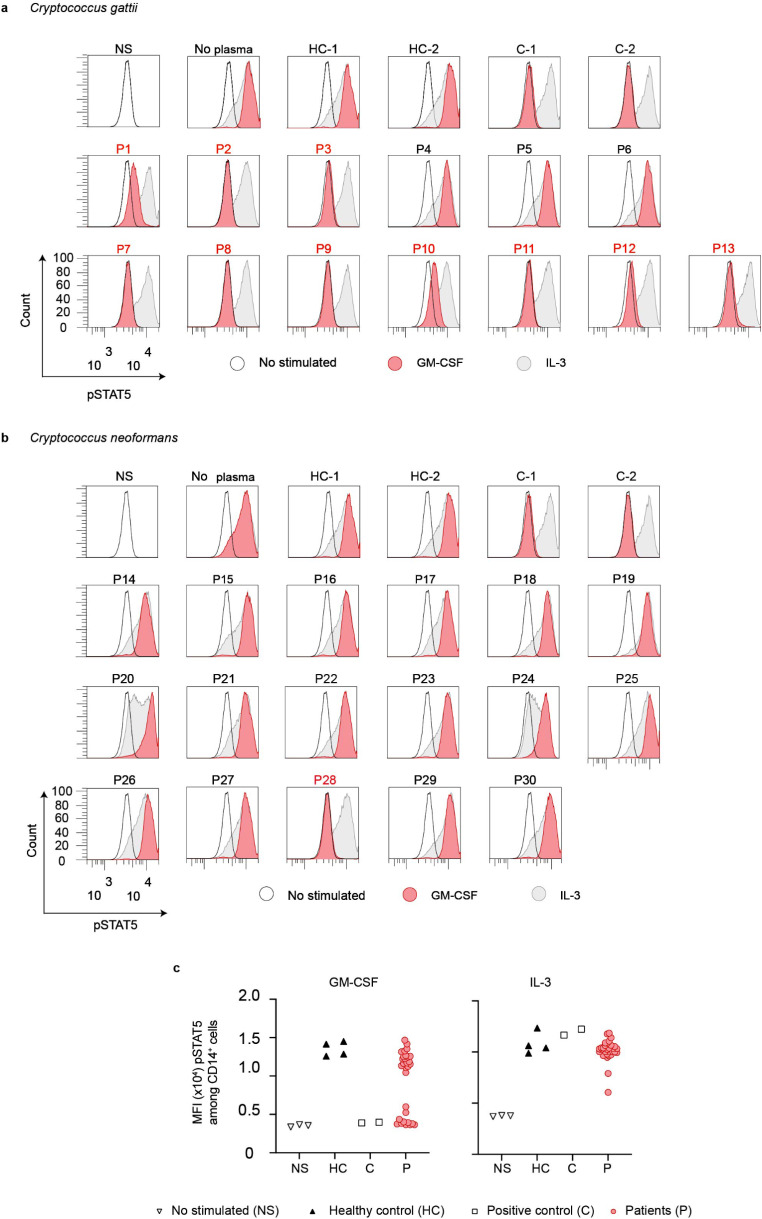
Neutralizing anti GM-CSF auto-Abs in patients with cryptococcosis caused by *Cryptococcus gattii or Cryptococcus neoformans*. Monoparametric histogram showing the STAT5 phosphorylation (pSTAT5), as assessed by flow cytometry, upon the stimulation with recombinant human (rh)GM-CSF (red) or rhIL-3 (grey) of control PBMCs, in the absence of serum, or in the presence of a 1:10 dilution of serum from two healthy individuals (HC-1 and HC-2), from two individuals previously described to carry neutralizing auto-Abs against GM-CSF (C-1 and C-2) or from thirty patients with cryptococcosis (P1 to P30). **a** Patients with cryptococcosis caused by *C. gattii* or, **b** patients with cryptococcosis caused by *C. neoformans*. Not stimulated (NS) PBMCs were used as the basal STAT5 phosphorylation (black line). **c** Dot Plot showing p-STAT5 mean fluorescence intensity (MFI) of PBMCs, NS or stimulated with rhGM-CSF or rhIL-3 in the presence of 10% of plasma from healthy controls, individuals with or without anti-GM-CSF auto-Abs from **a** and **b**. HC, healthy controls; C, positive control for anti-GM-CSF neutralizing auto-Abs; P, patients with cryptococcosis; NS, not stimulated. The figure is representative of three independent experiments

We incubated PBMCs from healthy controls with 10 ng/mL of GM-CSF or IL-3 in the presence of 10% of plasma from healthy individuals or from the 30 HIV-negative patients with cryptococcosis. A block of STAT5 phosphorylation was observed in 10 patients (P1-P3, P7-P13) infected by *C. gattii* previously tested positive for the presence auto-Abs against GM-CSF (Fig. 2a, c). We also observed a complete abolition of STAT5 phosphorylation in one patient (P28) out of two infected by *C. neoformans,* previously tested positive for the presence auto-Abs against GM-CSF. In contrast, the serum from patient P22, despite tested positive for the presence of anti-GM-CSF IgG, was not able to neutralize GM-CSF in vitro (Fig. 2b, c). The levels of IL-3-induced p-STAT5 were similar between cells incubated with controls’ or patients’ sera (Fig. 2a, b and c). Overall, we found that 10 of 13 patients (77%) infected by *C. gattii*, and 1 of 17 patients (6%) infected by *C. neoformans* had neutralizing IgG auto-Abs against GM-CSF. One serum sample (P22, 6%) of the 17 patients infected by *C. neoformans*, tested positive for the presence IgG against GM-CSF, was not neutralizing in vitro. Taken together, these results strongly suggest that the presence of circulating neutralizing auto-Abs against GM-CSF is a risk factor for the development of cryptococcosis in 11 of the studied patients.

### Clinical and Immunological Data in Patients with Neutralizing Auto-Abs Against GM-CSF

Among the 30 patients studied, 10 (76.9%) with cryptococcosis caused by *C. gattii* and one (6%) with cryptococcosis caused by *C. neoformans*, were positive for neutralizing auto-Abs against GM-CSF. The demographic, clinical and microbiological characteristics of both groups (patients who were positive and negative for neutralizing auto-Abs against GM-CSF) were compared (Table 1). Patients with neutralizing auto-Abs against GM-CSF were predominantly affected by *C. gattii*. Sex, age, and clinical presentation did not differ among patients with and without auto-Abs. The clinical characteristics of patients with cryptococcosis, with and without neutralizing auto-Abs against GM-CSF, are summarized in supplementary Tables 2 and 3. Total levels of immunoglobulins were determined; however, the total levels of IgG, IgM and IgA did not differ between patients with or without neutralizing auto-Abs against GM-CSF or between those infected by *C. neoformans* or *C. gattii* (Supplementary Fig. 1a, b and c). Compared to healthy controls, we observed higher levels of total IgG in patients with cryptococcosis, whereas the total IgA and IgM levels were lower. Nevertheless, sera from patients with cryptococcosis caused by *C. gattii* and neutralizing auto-Abs against GM-CSF showed higher levels of specific IgG against cryptococcal proteins compared to healthy controls, as previously reported (Supplementary Fig. 1d and e) [22].

**Table 1:** Characteristics of patients with cryptococcosis with (+) and without (-) auto-Abs against GM-CSF

Demographic, clinical, and microbiological variables	Neutralizing auto-Abs against GM-CSF (+) (*n* = 11)	Neutralizing auto-Abs against GM-CSF (-) (*n* = 19)	*p*-value^1^
Male/Female ratio	8:3	13:6	1
Male	72.7%	68.4%	1
Age: mean – extreme values (years)	39.9 (23–67)	41.3 (1–71)	
Clinical presentation			
CNS involvement	9 (81.8%)	17 (89.5%)	0.5837
Lung involvement	1 (9.1%)	2 (10.5%)	1
CNS + lung involvement^2^	1 (9.1%)	0	0.3293
Risk factor non-HIV			
Unknown	11 (100%)	14 (73.7%)	0.1394
Hematological malignancy	0	2 (10.5%)	0.5477
Autoimmune disease (SLE, RA)^3^	0	2 (10.5%)	0.5477
Solid neoplasm	0	1 (5.3%)	1
Treatment			
AMBd + 5 FC^4^	2 (18.2%)	0	0.09145
AMBd + FLC^4^	6 (54.5%)	2 (10.5%)	0.07396
AMBd^4^	2 (18.2%)	12 (63.2%)	0.05397
No data	1 (9.1%)	5 (26.3%)	0.6582
Outcome			
Cure	6 (54.5%)	2 (10.5%)	0.005997
Deceased	1 (9.1%)	2 (10.5%)	1
No data	4 (36.4%)	15 (78.9%)	0.01799
Proven cryptococcosis			
Culture	11 (100%)	19 (100%)	NA
Species complex			
*C. gattii*	10 (90.9%)	3 (15.8%)	0.0009995
*C. neoformans*	1 (9.1%)	16 (84.2%)	0.0004998
Molecular types			
VNI	1 (9.1%)	16 (84.2%)	0.0004998
VGI	1 (9.1%)	0	0.3293
VGII	7 (63.6%)	2 (10.5%)	0.002499
VGIII	2 (18.2%)	1 (5.3%)	1
Serotype			
A	1 (9.1%)	16 (84.2%)	0.0004998
B	10 (90.9%)	2 (10.5%)	0.0004998
C	0	1 (5.3%)	1
D	0	0	NA

^1^(p-value calculated with a chi-square test with Fisher’s correction given the low n in some of the cells of the contingency tables)

^2^Pulmonary cryptococcoma with positive culture and histopathology results

^3^SLE=systemic lupus erythematosus, RA = rheumatoid arthritis

^3^AMBd= amphotericin B deoxycholate, 5FC = 5-flucytosine, FLC = fluconazole

### The Phenotypic and Genotypic Characteristics of Cryptococcal Isolates do not Differ Among Patients with and without Neutralizing Auto-Abs Against GM-CSF

All *C. gattii* isolates that caused cryptococcosis in patients with or without neutralizing auto-Abs against GM-CSF, with antifungal susceptibility test data available, were distributed within the wild-type population of the species for each antifungal drug tested (Supplementary Tables 4 and 5). This indicates that none of the isolates showed resistance or decreased susceptibility to any of the antifungal drugs tested. In addition, *C. gattii* isolates recovered from patients with or without neutralizing auto-Abs against GM-CSF did not differ in terms of serotype, mating type, molecular type, or ST of the isolates (Supplementary Fig. 1f). Similarly, the serotype, mating type and molecular type also did not differ among the *C. neoformans* isolates.

## Discussion

Our study reports the presence of neutralizing auto-Abs against GM-CSF in 11 out of 30 HIV-negative Colombian adult patients who developed cryptococcosis, despite being considered immunocompetent based on their clinical history at the time of diagnosis [18]. None of the patients under investigation had a history of PAP and, like in previous studies, all 11 patients with neutralizing auto-Abs against GM-CSF described here were adults (23 to 67 years old). We showed that these auto-Abs were more prevalent in patients with *C. gattii*-induced cryptococcosis (auto-Abs present in 10 out of 13 patients, 77%) than in those with *C. neoformans-*induced cryptococcosis (1 out of 17 patients, 6%), highlighting additional differences in the epidemiology of cryptococcosis caused by these two species complexes [3, 4]. This also highlights neutralizing auto-Abs against GM-CSF as a significant risk factor for cryptococcosis especially due to *C. gattii*. Notably, the disease-causing factors of the remaining 19 patients with cryptococcosis (3 due to *C. gattii* and 16 due *C. neoformans*) without neutralizing auto-Abs against GM-CSF remain unknown. Inborn errors of the GM-CSF pathway may explain some of these cases.

Tuberculosis is endemic in Colombia [23]. Interestingly, one patient described here also developed Tb caused by *Mtb* one year after being diagnosed with disseminated cryptococcosis due to *C. neoformans*. To our knowledge, this is the third adult patient reported with disseminated cryptococcosis and neutralizing auto-Abs against GM-CSF who has developed Tb [13, 20]. Together with mouse and ex vivo studies on human monocyte-derived macrophages [24–26], our data suggest that intact GM-CSF signaling is crucial for the proper function of alveolar macrophage in mediating immunity to *Cryptococcus* spp. and possibly *Mtb*-related lung diseases in humans.

Whether these auto-Abs were present before cryptococcal diseases and remained silent until disease onset is unknown. Recent studies have demonstrated the causality of auto-Abs against cytokines as a main risk factor for specific infectious diseases [27, 28]. For example, preexisting neutralizing auto-Abs against type I IFNs were shown to be significant risk factors for several severe viral diseases such as life-threatening COVID-19 pneumonia (15–20%) [27], severe influenza pneumonia (5%) [29], adverse reactions to live-attenuated yellow fever virus vaccine (30%) [28], or West Nile virus encephalitis (40%) [30]. These auto-Abs are present in about 0.3% of individuals of the general population under the age of 65 years, with prevalence increasing to at least 4% after the age of 70 years [28, 30]. However, the actual prevalence of neutralizing auto-Abs against GM-CSF in patients with cryptococcosis and in healthy individuals from the general population remains unknown.

The epidemiology of cryptococcosis has primarily focused on patients with HIV, the major risk factor for the disease [31]. However, recent studies on cryptococcosis have highlighted an increase of cases among HIV-negative individuals who appear otherwise healthy [32–35]. In Colombia, data from the National Surveillance on cryptococcosis (1997 to 2016) showed that 75.4% of 1974 patients included were HIV-positive. Among 392 patients HIV-negative, 51 (13%) had cryptococcosis due to *C. gattii* [18]. In Brazil, HIV remains the main risk factor for cryptococcosis (82–86%) [36, 37]. Similarly to what is observed in Colombia, among 29 HIV-negative Brazilian patients, most were infected by *C. gattii* [38]. In French Guiana, HIV is also the main risk factor for cryptococcosis (67.4%). This country also reported the first two cases of otherwise healthy Latin-American patients with cryptococcosis due to *C. gattii* and neutralizing auto-Abs against GM-CSF [39].These observations indicate a notable increase in cryptococcosis cases among non-HIV individuals, suggesting that other risk factors, such as neutralizing auto-Abs against GM-CSF, contribute to the disease susceptibility.

Our findings support the hypothesis and emerging research suggesting that auto-Abs against cytokines contribute to susceptibility to specific infectious diseases in otherwise healthy individuals [5, 8]. However, the laboratory techniques commonly used to detect these auto-Abs in a clinical setting are often expensive or not readily available, particularly in underdeveloped countries where diseases like cryptococcosis are endemic [18]. Additionally, technologies such as flow cytometry and particle-based assays, used to screen for anti-cytokines auto-Abs, are primarily utilized as research tools rather than standard diagnostic methods for infectious diseases [13, 14, 20, 40]. Lastly, these approaches only assess the presence or absence of auto-Abs, but they do not evaluate their neutralization activity. Therefore, it is crucial to advance the development of accessible diagnostic methods within healthcare settings.

## Conclusions

This study supports the well-established association between neutralizing anti-GM-CSF auto-Abs and susceptibility to cryptococcosis; though, the timing and mechanisms of their appearance remain unknown. It is also unknown why patients with anti-GM-CSF auto-Abs are more susceptible to *C. gattii* than *C. neoformans* cryptococcosis. The presence of these auto-Abs in individuals with pulmonary and/or meningeal cryptococcosis underscores the critical role of GM-CSF in host defense against *Cryptococcus* spp. Our results strongly suggest that unexplained cryptococcosis in otherwise healthy individuals should prompt the search for neutralizing anti-GM-CSF auto-Abs, especially in endemic countries.

### Electronic Supplementary Material

Below is the link to the electronic supplementary material.

Supplementary Material 1

## Data Availability

All data are either included in the manuscript or available upon request.
