# Supplementary material for: Autoantibodies Neutralizing GM-CSF in HIV-Negative Colombian Patients Infected with Cryptococcus gattii and C. neoformans
Source: J Clin Immunol. 2024 Jul 15;44(7):163. doi: 10.1007/s10875-024-01757-y (PMC11249431; doi:10.1007/s10875-024-01757-y)
Supplement: Supplementary file 1 — Supplementary Material 1 [file 10875_2024_1757_MOESM1_ESM.docx]

**Supplementary information**

**Autoantibodies neutralizing GM-CSF in HIV-negative Colombian patients infected with *Cryptococcus gattii* and *C. neoformans***

Carlos A. Arango-Franco^1,2,+, &^, Julian Rojas^1,+^,Carolina Firacative^3^, Mélanie Migaud^2^, Clara Inés Agudelo^4^, José Luis Franco^1^, Jean-Laurent Casanova^2,5,6,7,8^, Anne Puel^2,5,6,*^, Jairo Lizarazo^9,*^, Elizabeth Castañeda^4,*^, and Andrés A. Arias^1,10,*, &^

^1^ Group of Inborn Errors of Immunity. Department of Microbiology and Parasitology. School of Medicine, University of Antioquia (UdeA), Medellín, Colombia.

^2^ Laboratory of Human Genetics of Infectious Diseases. Necker Branch, INSERM U1163, Necker Hospital for Sick Children. Paris, France.

^3^ Studies in Translational Microbiology and Emerging Diseases (MICROS) Research Group, School of Medicine and Health Sciences, University del Rosario, Bogotá, Colombia.

^4^ Microbiology Group, Instituto Nacional de Salud, Bogotá, Colombia.

^5^ University of Paris Cité, Imagine Institute. Paris, France.

^6^ St. Giles Laboratory of Human Genetics of Infectious Diseases, Rockefeller Branch, The Rockefeller University. New York, NY, USA.

^7^ Howard Hughes Medical Institute, New York, NY, USA

^8^ Pediatric Hematology and Immunology Unit, Necker Hospital for Sick Children, Paris, France.

^9^ Internal Medicine Department, Hospital Universitario Erasmo Meoz, University of Pamplona. Cúcuta, Colombia.

^10^ School of Microbiology, University of Antioquia (UdeA), Medellin, Colombia.

^+^ and * These authors contributed equally.

^&^Correspondence: [aaugusto.arias@udea.edu.co](mailto:aaugusto.arias@udea.edu.co), carlos.arango2@udea.edu.co

**Survey description. Ethical Considerations**

**Supplementary table 1**. Characteristics of cryptococcosis patients studied for auto-Abs against GM-CSF

**Supplementary table 2.** Clinical characteristics of patients with cryptococcosis and GM-CSF auto-Abs

**Supplementary table 3.** Clinical characteristics of patients with cryptococcosis and negative for GM-CSF auto-Abs

**Supplementary table 4.** Microbiological characteristics of patients with cryptococcosis and GM-CSF auto-Abs

**Supplementary table 5.** Microbiological characteristics of patients with cryptococcosis and negative for GM-CSF auto-Abs

**Supplementary figure 1.** Total and specific immunoglobulin levels in patients with cryptococcosis with and without neutralizing auto-Abs against GM-CSF and relationship between *Cryptococcus gattii* and *C. neoformans* strains isolated from patients

**Supplementary figure 2.** Neutralizing assay gating strategy

**References**

**Survey description**

In Colombia, a laboratory-based surveillance system has been applied passively and voluntarily since 1997 and has aimed to determine the demographic characteristics of patients, risk factors associated with the disease, laboratory diagnoses, signs and symptoms, treatments, types of cryptococcosis and outcomes. The survey was administered between 1997 and the present. The survey was designed in 1997 and updated in 2012 following the guidelines established by the European Confederation of Medical Mycology with the corresponding authorization. Each format of the survey was previously filled out by health professionals attending patients from public and private institutions, as well as from the public health laboratories of the Colombian political divisions (departments). The survey is divided into eight sections, which included the following pieces of information: demographic data, risk factors and whether cryptococcosis was an aids-defining illness or not, diagnosis, diagnostics images, clinical manifestations, cryptococcosis classification documented in the survey (pulmonary, meningitis, cutaneous, bone, disseminated or infection), and treatment with other therapies. Considering the survey does not include a follow-up of the patients, we focus exclusively on the clinical data that was collected at the moment of the survey.

Complete surveys and isolates or samples were sent to the study coordinating center (Instituto Nacional de Salud in Bogotá), and a database was created.

**Ethical considerations**

The present study endorsed of the ethics committee of the Instituto Nacional de Salud, and its implementation was subjected to the principles of medical research in human beings stated in the Helsinki Declaration. Although this survey is designed to be descriptive, patient identification was completely anonymized. No additional tests were needed beyond those required by the consulting physician.

**Supplementary table 1**. Characteristics of cryptococcosis in patients studied for neutralizing auto-Abs against GM-CSF (n=30)

| **Patient** | **Survey/**  **year dx** | **Age (years)** | **Sex** | **Origin** | **Presence of GM-CSF auto-Abs** | **Neutralization assay *in vitro*** |
| --- | --- | --- | --- | --- | --- | --- |
| ***C. gattii*** | | | | | | |
| P1 | 12/1997 | 36 | F | Caquetá | + | + |
| P2 | 101/1997 | 23 | M | Bolívar | + | + |
| P3 | 103/1998 | 30 | M | Bogotá | + | + |
| P4 | 222/2001 | 29 | M | Norte de Santander | - | - |
| P5 | 231/2001 | 39 | M | Norte de Santander | - | - |
| P6 | 468/2002 | 56 | M | Norte de Santander | - | - |
| P7 | 472/2002 | 28 | M | Norte de Santander | + | + |
| P8 | 506/2002 | 32 | M | Meta | + | + |
| P9 | 521/2002 | 67 | M | Bogotá | + | + |
| P10 | 615/2003 | 54 | M | Bogotá | + | + |
| P11 | 636/2003 | 54 | M | Norte de Santander | + | + |
| P12 | 752/2004 | 46 | M | Caquetá | + | + |
| P13 | 1554/2011 | 29 | F | Norte de Santander | + | + |
| ***C. neoformans*** | | | | | | |
| P14 | 300/2000 | 6 | M | Bogotá/Casanare | - | - |
| P15 | 333/2000 | 1 | M | Norte de Santander | - | - |
| P16 | 270/2001 | 41 | M | Norte de Santander | - | - |
| P17 | 271/2001 | 14 | F | Norte de Santander | - | - |
| P18 | 381/2001 | 71 | F | Bogotá | - | - |
| P19 | 382/2001 | 66 | M | Bogotá | - | - |
| P20 | 420/2002 | 49 | M | Huila | - | - |
| P21 | 482/2002 | 22 | F | Bogotá | - | - |
| P22 | 495/2002 | 71 | F | Bogotá | + | - |
| P23 | 953/2002 | 40 | M | Bogotá | - | - |
| P24 | 600/2003 | 71 | M | Bogotá/Cundinamarca | - | - |
| P25 | 950/2004 | 51 | M | Bogotá | - | - |
| P26 | 958/2005 | 68 | F | Norte de Santander | - | - |
| P27 | 877/2005 | 62 | F | Norte de Santander | - | - |
| P28 | 876/2005 | 40 | F | Norte de Santander | + | + |
| P29 | 1028/2006 | 6 | M | Bogotá/Cundinamarca | - | - |
| P30 | 1133/2007 | 22 | M | Bogotá/Cundinamarca | - | - |

Retrieved from (1, 2)

**Supplementary table 2.** Clinical characteristics of patients with cryptococcosis and neutralizing auto-Abs against GM-CSF (n=11)

| Patient | Symptoms and signs | Clinical form | Risk factor | Treatment^1^ | Outcome |
| --- | --- | --- | --- | --- | --- |
| P1 | ND | Meningitis | Unknown | ND | ND |
| P2 | Headache, fever | Meningitis | Unknown | AMBd + 5FC | Success |
| P3 | Headache | Meningitis | Unknown | AMBd + FLC | Deceased |
| P7 | Headache, vomiting, mental confusion, meningeal signs, loss of vision | Meningitis | Unknown | AMBd | Success |
| P8 | Fever, vomiting, mental confusion | Meningitis | Unknown | AMBd + 5FC | Success |
| P9 | Cough, fever, shortness of breath | Pulmonary | Unknown | AMBd + FLC | ND |
| P10 | Headache, vomiting, mental confusion, meningeal signs | Meningitis | Unknown | AMBd + FLC | ND |
| P11 | Headache, vomiting, mental confusion | Meningitis | Unknown | AMBd + FLC | Success |
| P12 | Headache, vomiting, mental confusion | Meningitis | Unknown | AMBd | ND |
| P13 | Headache, vomiting, mental confusion, papilledema, vision loss | Meningitis | Unknown | AMBd + FLC | Success^2^ |
| P28 | Headache, vomiting, meningeal signs | Pulmonary Cryptococcoma Meningitis | Unknown | AMBd + FLC | Success^3^ |

^1^AMBd: amphotericin B deoxycholate; FLC: fluconazole; 5FC: 5-flucytosine; ND: no data ^2^ significant vision loss^. 3^ Pulmonary tuberculosis was subsequently developed. Retrieved from (1).

**Supplementary table 3.** Clinical characteristics of patients with cryptococcosis who were negative for neutralizing auto-Abs against GM-CSF (n=19)

| Patient | Symptoms and signs | Clinical form | Risk factor | Treatment^1^ | Outcome |
| --- | --- | --- | --- | --- | --- |
| P4 | Headache, nausea, and fever | Meningitis | Unknown | AMBd | ND |
| P5 | Headache, nausea cough, abnormal vision, neurological focus | Meningitis | Unknown | AMBd | ND |
| P6 | Headache, nausea, and abnormal vision | Meningitis | Unknown | AMBd | Success |
| P14 | Headache, nausea, abnormal vision, and hydrocephalus | Meningitis | Unknown | ND | ND |
| P15 | Fever, confusion | Meningitis | Unknown | AMBd | ND |
| P16 | Headache, nausea, confusion, abnormal vision | Meningitis | Unknown | AMBd | ND |
| P17 | Headache, nausea, fever, confusion, and abnormal vision | Meningitis | Unknown | AMBd | ND |
| P18 | Headache, fever, cough | Meningitis | Lymphoma | ND | ND |
| P19 | Headache, nausea, fever, confusion, cough | Meningitis | Unknown | AMBd | ND |
| P20 | Headache, nausea, confusion, abnormal vision, and hydrocephalus | Meningitis | Unknown | ND | ND |
| P21 | Headache, nausea, fever, confusion and neurological focusing | Pulmonary | SLE | AMBd | Deceased |
| P22 | Headache | Meningitis | Hematological malignancy | ND | Success |
| P23 | None | Meningitis | Unknown | AMBd | ND |
| P24 | Fever | Meningitis | Hematological malignancy | ND | ND |
| P25 | None | Meningitis | Arthritis | AMBd | ND |
| P26 | Fever, nausea, confusion | Meningitis | Unknown | AMBd + FLC | ND |
| P27 | Headache, nausea, fever, confusion | Meningitis | Unknown | AMBd | Deceased |
| P29 | Headache, nausea, fever | Meningitis | Unknown | AMBd + FLC | ND |
| P30 | Palate injury | Pulmonary | Unknown | AMBd | ND |

^1^ AMBd: amphotericin B deoxycholate; FLC: fluconazole; ND: No data. Retrieved from (1, 2)

**Supplementary table 4.** Microbiological characteristics of patients with cryptococcosis and neutralizing auto-Abs against GM-CSF (n=11)

| Patient | Sample | | | | | | | MIC (µg/ml)^1^ | | | | | | | | Serotype, Mating type, Molecular. Type, ST^2^ |
| --- | --- | --- | --- | --- | --- | --- | --- | --- | --- | --- | --- | --- | --- | --- | --- | --- |
|  | Serum | | | CSF | | | |  |  |  |  |  |  |  |  |  |
|  | Code | CrAg | CrAg | | Direct examination | Strain code | 5FC | | POS | VRC | ITC | FLC | AMB |  | | |
|  |  | titer | titer | |  |  |  |  |  |  |  |  |  |  | | |
| P1 | S332-1997 | 1:2048 | 1:2048 | | + | 638 | 4 | | 0.25 | 0.25 | 0.125 | 16 | 0.5 | B, α, VGII, 321 | | |
| P2 | S290-1997 | ND^3^ | ND | | + | 675 | 1 | | 0.125 | 0.125 | 0.125 | 16 | 1 | B, α, VGII, 25 | | |
| P3 | S189-1998 | 1:2048 | 1:2048 | | + | 628 | 2 | | 0.125 | 0.0625 | 0.0625 | 4 | 0.25 | B, a, VGI, 51 | | |
| P7 | S93-2004 | ND | ND | | + | 1509 | ND | | | | | | | | B, α, VGIII, 146 | |
| P8 | S48-2002 | ND | 1:32 | | + | 1442 | ND | | | | | | | | B, α, VGII, 29 | |
| P9 | S208-2002 | 1:8 | ND | | ND^4^ | 1543 | 1 | | 0.125 | 0.125 | 0.0625 | 16 | 0.25 | B, α, VGII, 12 | | |
| P10 | S105-2003 | ND | 1:1024 | | + | 1708 | 0.5 | | 0.0625 | 0.0625 | 0.0625 | 8 | 0.25 | B, a, VGIII, 59 | | |
| P11 | S88-2005 | ND | 1:1 | | + | 1959 | 8 | | 0.125 | 0.0625 | 0.0625 | 8 | 0.5 | B, a, VGII, 46 | | |
| P12 | S129-2004 | 1:1 | 1:1 | | + | 2151 | ND | | | | | | | | B, a, VGII, 25 | |
| P13 | S102-2011 | ND | ND | | + | 3407 | 2 | | 0.0625 | 0.03125 | 0.03125 | 4 | 0.25 | B, a, VGII, 324 | | |
|  |  |  |  |  |  |  |  |  |  |  |  |  |  |  |  |  |
| P28 | S 295-2005 | ND | 1:1024 | | + | 2373 | ND | | | | | | | | A, ND, VNI, ND | |

^1^ Retrieved from (3) ^2^ST: Sequence type, ^3^ND: not done, ^4^Sputum sample

5FC: 5-flucytosine; POS: posaconazole; VRC: voriconazole; ITC: itraconazole; FLC: fluconazole; AMB: amphotericin B

**Supplementary table 5.** Microbiological characteristics of patients with cryptococcosis who were negative for neutralizing auto-Abs against GM-CSF (n=19)

| Patient | Survey code/year | Sample | | | | | | MIC (µg/ml)^1^ | | | | | | | | Serotype, Mating type, Molecular type, ST^2^ |
| --- | --- | --- | --- | --- | --- | --- | --- | --- | --- | --- | --- | --- | --- | --- | --- | --- |
|  |  | Serum | | CSF | | | |  |  |  |  |  |  |  |  |  |
|  |  | Code | CrAg | CrAg | Direct examination | Strain | 5FC | | POS | VRC | ITC | FLC | AMB |  | | |
|  |  |  | titer | titer |  | code |  |  |  |  |  |  |  |  | | |
| ***C. gattii*** | | | | | | | | | | | | | | | | |
| P4 | 222/2001 | S202-2001 | ND^3^ | ND | + | 1276 | ND | | | | | | | | C, α, VGIII, 64 | |
| P5 | 231/2001 | S255-2001 | ND | ND | + | 1278 | ND | | | | | | | | B, a, VGII, 25 | |
| P6 | 468/2002 | S318-2002 | ND | ND | + | 1511 | 2 | | 0.125 | 0.0625 | 0.0625 | 8 | 0.25 | B, a, VGII, 25 | | |
| ***C. neoformans*** | | | | | | | | | | | | | | | | |
| P14 | 300/2000 | S353-2000 | ND | 1:1 | + | 1163 | ND | | | | | | | | A, α, VNI | |
| P15 | 333/2000 | S144v2-2002 | ND | ND | + | 1153 | ND | | | | | | | | A, α, VNI | |
| P16 | 270/2001 | S145-2002 | ND | ND | + | 1192 | ND | | | | | | | | A | |
| P17 | 271/2001 | S42-2002 | ND | ND | + | 1193 | ND | | | | | | | | A, α, VNI | |
| P18 | 381/2001 | S2-2001 | ND | 1:1024 | + | 1182 | ND | | | | | | | | A | |
| P19 | 382/2001 | S144-2001 | ND | 1:1 | + | 1209 | ND | | | | | | | | A | |
| P20 | 420/2002 | S9-2002 | ND | + | + | 1402 | ND | | | | | | | | A | |
| P21 | 482/2002 | S198-2002 | ND | ND | ND^4^ | 1554 | ND | | | | | | | | A | |
| P22 | 495/2002 | S193-2002 | ND | ND | + | 1545 | ND | | | | | | | | A | |
| P23 | 953/2002 | S311-2002 | ND | 1:1 | + | 1592 | ND | | | | | | | | A | |
| P24 | 600/2003 | S57-2003 | ND | 1:1 | + | 1646 | ND | | | | | | | | A | |
| P25 | 950/2004 | S309-2004 | ND | 1:1 | + | 2265 | ND | | | | | | | | A | |
| P26 | 958/2005 | S387-2005 | ND | 1:1024 | + | 2451 | ND | | | | | | | | A | |
| P27 | 877//2005 | S58-2005 | 256 | 1:1024 | + | 2356 | ND | | | | | | | | A | |
| P29 | 1028/2006 | S186-2006 | ND | ND | + | 2621 | ND | | | | | | | | A, α, VNI | |
| P30 | 1133/2007 | 1S22-2007 | ND | ND | ND^5^ | 2802 | ND | | | | | | | | A, ND,VNI | |

^1^ Retrieved from (1-3); ^2^ST: Sequence type; ^3^ND: not done; ^4^ Blood sample; ^5^ Pharynx exudate.

5FC: 5-flucytosine; POS: Posaconazole; VRC: Voriconazole; ITC: Itraconazole; FLC: Fluconazole; AMB: Amphotericin B


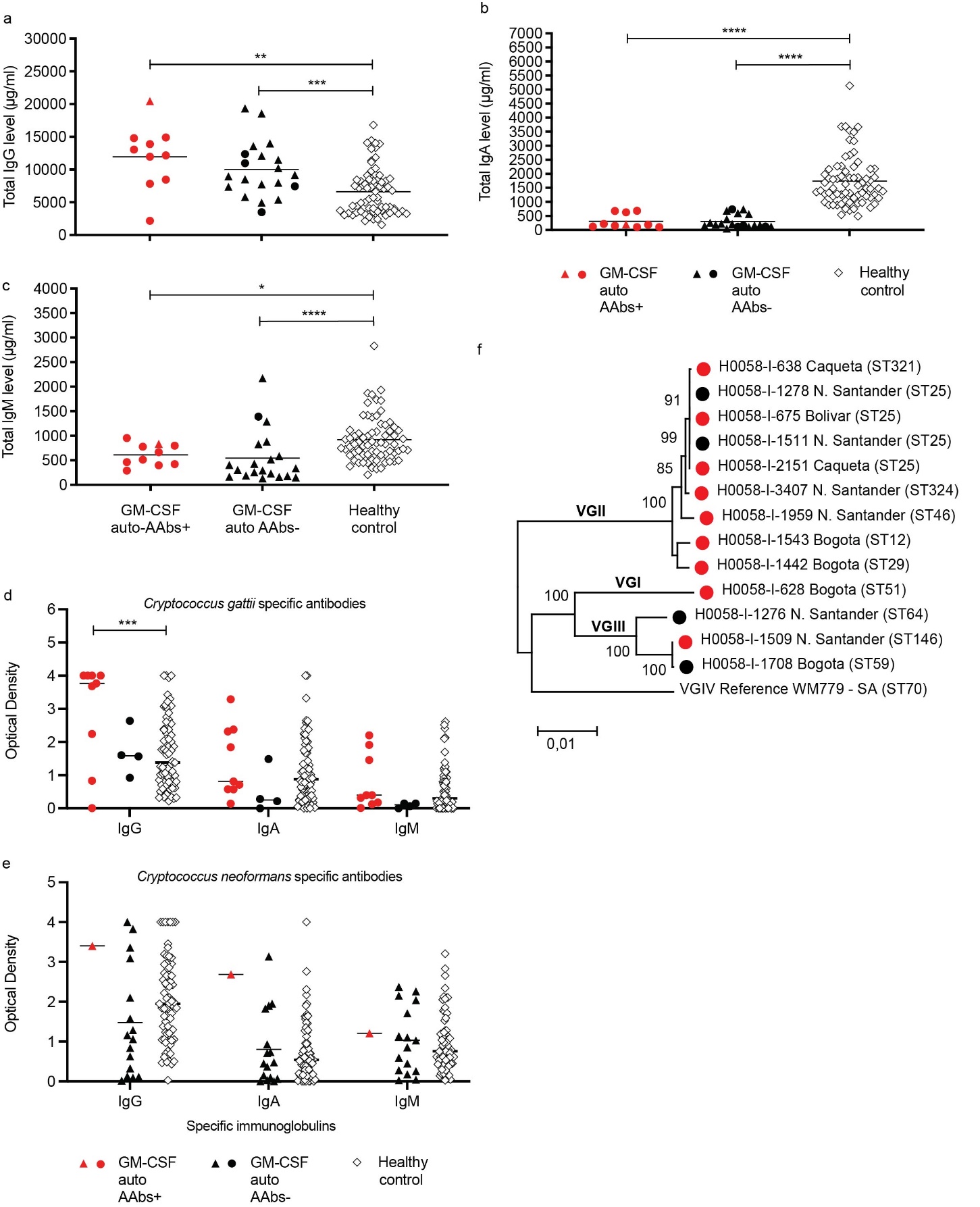


**Supplementary figure 1.** Total and specific immunoglobulin levels in patients with cryptococcosis with and without neutralizing auto-Abs against GM-CSF and the relationship between *Cryptococcus gattii* and *C. neoformans* strains isolated from patients.

Titers of serum IgG (**a**) IgA (**b**), and IgM (**c**) in patients caused by cryptococcosis by *Cryptococcus gattii* (○) or *C. neoformans* (△), and healthy controls without cryptococcosis (◊). Optical density of specific levels of serum IgG, IgA and IgM against *C. gattii* (**d**) and *C. neoformans* proteins (**e**). In figures **a-e**, each spot represents a serum sample of an individual. The median value is shown by a horizontal line. Mann-Whitney nonparametric test for unpaired samples was performed to determine statistical differences in the total (**a, b, c**) and specific levels of immunoglobulins (**d, e**) between groups. Dendrogram showing the genetic relationship between *C. gattii* isolates recovered from patients with cryptococcosis (**f**). The dendrogram was constructed with the software Mega version 11 based on maximum likelihood analysis. In all figures, serum samples or isolates from patients with neutralizing auto-Abs against GM-CSF are marked in red, individuals without neutralizing auto-Abs against GM-CSF in black, and healthy controls without cryptococcosis in white. Retrieved from (1, 2, 4).


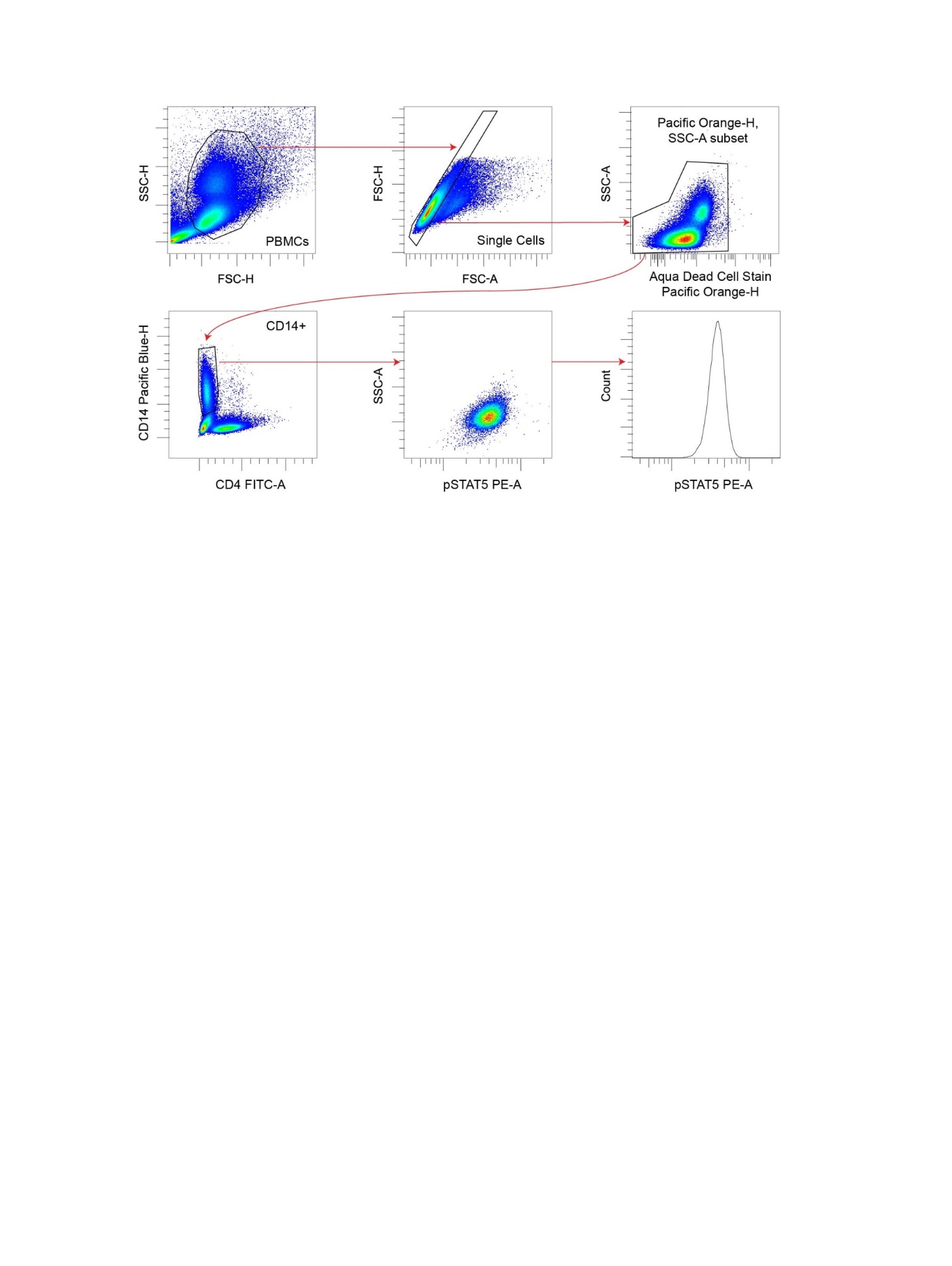


**Supplementary figure 2.** Neutralizing assay gating strategy. Strategy for analysis of flow cytometry data from neutralizing auto-Abs against GM-CSF assay performed on serum of the patients. The analysis was performed in Flowjo V10

**References**

1. Lizarazo J, Escandón P, Agudelo CI, Firacative C, Meyer W, Castañeda E. Retrospective study of the epidemiology and clinical manifestations of *Cryptococcus* *gattii* infections in Colombia from 1997-2011. PLoS Negl Trop Dis. 2014;8(11):e3272.

2. Escandon P, Lizarazo J, Agudelo CI, Castaneda E. Cryptococcosis in Colombia: Compilation and analysis of data from laboratory-based surveillance. J Fungi (Basel). 2018;4(1).

3. Firacative C, Escandón P. Antifungal susceptibility of clinical *Cryptococcus gattii* isolates from Colombia varies among molecular types. Med Mycol. 2021;59(11):1122-5.

4. Becerra-Álvarez P, Escandón P, Lizarazo J, Quirós-Gómez Ó, Firacative C. *Cryptococcus neoformans* and *Cryptococcus gattii*-specific IgG, IgA and IgM differ among children and adults with and without cryptococcosis from Colombia. Med Mycol. 2022;60(9).
